# ANALYSIS OF IMPACT OF DAILY OBJECTS’ SHAPE ON STANDING AND SITTING BEHAVIOR OF FRAIL OLDER PEOPLE

**DOI:** 10.1093/geroni/igae098.0494

**Published:** 2024-12-31

**Authors:** Satoru Handa, Koji Kitamura, Hisashi Kawai, Yoshifumi Nishida

**Affiliations:** Tokyo Institute of Science Tokyo, Meguro City, Tokyo, Japan; National Institute of Advanced Industrial Science and Technology, Tokyo, Japan; Tokyo Metropolitan Institute for Geriatrics and Gerontology, Tokyo, Japan; Tokyo Institute of Science Tokyo, Meguro City, Tokyo, Japan

## Abstract

One promising approach to supporting the independent lives of frail older people is the design of living environments. The objective of this study is to clarify the effect of slight changes in the three-dimensional shape of daily environments, such as a desk, on standing and sitting by conducting experiments on frail older people. To this end, the authors fabricated four types of desks with different shapes and then measured the standing and sitting movements of frail older people (N=85, 65 to 90 years old) using the desks, a motion capture system, three-axis force sensors, and pressure sensors. The measure data were analyzed from psychological and physical viewpoints. Eight of the 85 applicants had severe sarcopenia. The results showed that handrails and fingerholds significantly increased the force to hold the desk (p< 0.0001), and analysis using a musculoskeletal model (AnyBody) revealed that they decreased the muscle activity to push the desk (p=0.015).The cluster analysis results using posture data present that the handrails and handholds of the desk contributed to shifting the center of gravity of the upper body forward, leading to a decreasing burden on the muscles. A post-measurement questionnaire indicated that over 80% of the participants felt desks with handrails or handholds were more accessible to support and stand on. Some participants commented that the slight shape change improved a sense of safety. These results suggest that even minor design features such as handrails and fingerholds can assist the older in shifting their upper body posture without increasing the muscles’ burden.

